# Angiotensin-converting enzyme inhibitors (ACEI) or angiotensin receptor blockers (ARBs) may be safe for COVID-19 patients

**DOI:** 10.1186/s12879-021-05821-5

**Published:** 2021-01-25

**Authors:** Wenjun Wang, Xiaohui Zhao, Wei Wei, Weiwang Fan, Kai Gao, Shengxiu He, Xijing Zhuang

**Affiliations:** 1 The Department of Cardiovascular Surgery, Dalian Municipal Central Hospital, Dalian Medical University, Dalian, China; 2 Depart of Respiratory Medicine, Dalian Municipal Central Hospital, Dalian, China; 3 Department of Neurosurgery, Dalian Municipal Central Hospital, Dalian, China; 4 Department of Emergency Medicine, Dalian Municipal Central Hospital, Dalian, China; 5 Department of Intensive Care Unit, Dalian Municipal Central Hospital, Dalian, China; 6 Department of Oncology, Dalian Municipal Central Hospital, Dalian, China

**Keywords:** Hypertension, Renin-angiotensin-aldosterone system, Angiotensin-converting enzyme inhibitor (ACEI), Angiotensin receptor blockers (ARBs), COVID-19

## Abstract

**Background:**

To investigate the effects of angiotensin-converting enzyme inhibitor (ACEI) and angiotensin receptor blockers (ARBs) administration to hypertension patients with the coronavirus disease 2019 (COVID-19) induced pneumonia.

**Methods:**

We recorded the recovery status of 67 inpatients with hypertension and COVID-19 induced pneumonia in the Raytheon Mountain Hospital in Wuhan during February 12, 2020 and March 30, 2020. Patients treated with ACEI or ARBs were categorized in group A (*n* = 22), while patients who were not administered either ACEI or ARBs were categorized into group B (*n* = 45). We did a comparative analysis of various parameters such as the pneumonia progression, length-of-stay in the hospital, and the level of alanine aminotransferase (ALT), serum creatinine (Cr), and creatine kinase (CK) between the day when these patients were admitted to the hospital and the day when the treatment ended.

**Results:**

These 67 hypertension cases counted for 33.17% of the total COVID-19 patients. There was no significant difference in the usage of drug treatment of COVID-19 between groups A and B (*p* > 0.05). During the treatment, 1 case in group A and 3 cases in group B progressed from mild pneumonia into severe pneumonia. Eventually, all patients were cured and discharged after treatment, and no recurrence of COVID-2019 induced pneumonia occurred after the discharge. The length of stays was shorter in group A as compared with group B, but there was no significant difference (*p* > 0.05). There was also no significant difference in other general parameters between the patients of the groups A and B on the day of admission to the hospital (*p* > 0.05). The ALT, CK, and Cr levels did not significantly differ between groups A and B on the day of admission and the day of discharge (*p* > 0.05).

**Conclusions:**

To treat the hypertension patients with COVID-19 caused pneumonia, anti-hypertensive drugs (ACEs and ARBs) may be used according to the relative guidelines. The treatment regimen with these drugs does not need to be altered for the COVID-19 patients.

## Background

Coronavirus disease 2019 (COVID-19), caused by a novel coronavirus (SARS-CoV-2), began in Wuhan, Hubei province, China in December 2019. With a plethora of cases in almost every country, this disease has now put the doctors worldwide on alert [1]. In January 2020, the World Health Organization (WHO) emergency committee declared COVID-19 as a global health emergency in response to the growing case notification rate in China and other countries. The case detection rate that is changing every day can be tracked in real time on the Internet [2].

Similar to the SARS viruse, the SARS-CoV-2 infect alveolar cells via binding to the S-receptor binding domain and angiotensin-converting enzyme (ACE) 2 receptor and causes a series of pathological changes in the patients [3–9].

Both ACE and ACE2 are not only essential enzymes of the renin-angiotensin-aldosterone system (RAAS) but also important for the regulation of systemic blood pressure. ACE and ACE2 have the opposite effect and are widely involved in the regulation of physiological environment and functions, especially circulatory system function [10–12]. ACE increases blood pressure by increasing the circulation of angiotensin II (AngII), whereas ACE2 decreases blood pressure by lowering AngII [10–12].

Previous studies on SARS-CoV have shown that the elevation of AngII contributes to the occurrence and aggravation of acute pneumonia, and that SARS-CoV infection induces a decrease in the expression of tissue ACE 2 [13]. Based on these observations, researchers have advocated the use of RAS inhibitors such as ACEI or ARBs for alleviating pneumonia injuries induced by SARS-CoV-2 [14]. However, other studies have raised concerns while showing that some RAS inhibitors increase ACE2 expression [15]; therefore, the use of RAS inhibitors in COVID-19 patients may aggravate the disease [16, 17].

Thus, we paid special notice in the effects of ACEI or angiotensin receptor blockers (ARBs) in the progression and treatment of the COVID-19. As cardiovascular physicians, the authors participated in the treatment of COVID-19 patients in Raytheon Mountain Hosptial of Wuhan during the disease prevalence and focused on the treatment of hypertension in patients with pneumonia caused by COVID-19. In this study, we evaluated the therapeutic effect of an ACEI and ARBs on 67 inpatients with hypertension and COVID-19 caused pneumonia in the Raytheon Mountain Hospital in Wuhan between February 12, 2020 and March 30, 2020 and report their disease progression and recovery status.

## Methods

### Study design and ethics approval

This study is a retrospective study. This study was approved by the Ethics Committee of Dalian Central Hospital provided. The ethical approval number is YN-2020-023-01.

### Clinical setting and participants

The Raytheon Mountain Hospital in Wuhan has a total of 1600 beds, including 2 intensive care units (ICUs), 3 sub-ICUs and 27 general units. The hospital provides in-patient treatment for COVID-19 patients. The data included in this study were collected from COVID-19 patients hospitalized in A4, A8, A13, and B1 wards of Raytheon Mountain Hospital in Wuhan from February 18, 2020 to March 30, 2020.

We examined the disease progression and recovery status of patients with hypertension and COVID-19 induced pneumonia.

Our diagnostic criteria for hypertension was in accordance with the diagnostic criteria mentioned in “China Hypertension Prevention and Treatment Guidelines, 2018 revision” issued by the China Hypertension Prevention and Treatment Guidelines Revision Committee [18].

### Variables

The parameters measured in this study included age, gender, comorbidities, the severity classification and recovery status of COVID-19 induced pneumonia, COVID-19 medication, the medication used for hypertension, length-of-stay in the hospital, and serum level of ALT, Cr, and CK on the day of admission in the hospital (beginning of treatment) and the day of discharge from the hospital (end of the treatment).

The classification of severity of COVID-19 was based on the “The Diagnosis and Treatment Consensus of New Coronavirus Pneumonia ,Version 5th-7th”. According to the severity of the disease, patients are divided into mild, common, severe and critical [17, 19]. However, according to the requirements of our research, both mild and common types are classified as mild. Therefore, in our study, patients are classified into mild, severe, and critical due to their condition (Table 1).

Comorbidities include coronary heart disease, diabetes, liver cirrhosis, biliary tract infection, alzheimer’s disease, and nasopharyngeal cancer. The types of COVID-19 are: normal, severe and critical types [17, 19]. Drug treatment of COVID-19 mainly includes Abidor, Moxifloxacin, Lianhua Qingwen capsule and Traditional Chinese Medicine [17, 19].

Hypertension drugs used for all patients were calcium antagonists and β-receptor blockers. Calcium antagonist used in the treatment was either nifidipine 30 mg orally daily,or amlodipine 5 mg orally daily, and β-receptor blocker used was either metoprolol 25 mg orally twice a day,or bisoprolol 5 mg orally daily. Similarly, ARB given to group A patients was either valsartan 80 mg orally daily or irbesartan 150 mg orally daily, and ACEI given was benazepril 5 mg roally daily. The blood pressure of all the patients was kept under 140/90 mmHg with antihypertensive therapy [18].

Clinical outcome (death, recovery): COVID-19 recovery: discharge after treatment, in accordance with the discharge requirements of the seventh edition of novel coronavirus pneumonia treatment protocol [18]. Death: all causes of death.

### Data source

Data source was the hospital electronic health records.

### Measurements

Baseline data included age, gender, underlying disease, serum biochemical markers, and COVID-19 testing during the first 3 days after admission.

The data at discharge time included the data of the last medical record within one week before the discharge, including the drugs used for COVID-19, serum level of alanine aminotransferase (ALT), serum creatinine (Cr), creatine kinase (CK), disease changes and clinical outcome.

### Statistical analysis

All the clinical data of the patients were stored in Office 365. Statistical analyses were performed using SPSS Version 26.0 (IBM Corp. Released 2019, IBM SPSS Statistics for Windows, Armonk, NY, USA). Measurement data are represented as mean ± standard deviation. The measurement data that conformed to homogeneity of variance and normality were tested by student’s t-test. Wilcoxon rank-sum test was used for measurement data that did not conform to homogeneity and normality of variance. The counting data is expressed as the number of cases or composition ratio, and the comparison was performed using the χ2 test. For the expected frequency of a cell greater than 1 and less than 5, the continuous correction χ2 test was used. Fisher’s exact test was used for measurement data with the expected frequency of any cell is less than 1. The Kruskal-Willis test was used to compare multiple groups of data sets. All the tests were two-tailed, and the *P*-value less than 0.05 (*p* < 0.05) was considered statistically significant.

## Results

### Baseline data

A total of 202 patients (106 males and 96 females, with an average age of 60.00 ± 14.60 years) were hospitalized in wards A4, A8, A13, and B1 of Raytheon hospital since February 18, 2020 on March 30, 2020 (Table 2).

**Table 1:** The classification of severity of COVID-19 was based on the “The Diagnosis and Treatment Consensus of New Coronavirus Pneumonia ,Version 5th-7th”

Classification in this study	Classification by Chinese consensus	Description
Mild	Mild	With mild clinical symptoms, and there is no imaging finding of pneumonia.
	Common	With fever, respiratory symptoms, and imaging findings of pneumonia
Severe	Severe	Meet any of the following conditions: 1.Respiratory distress, RR ≥ 30 BPM 2.In resting state, finger pulse oxygen saturation ≤ 93% 3.arterial partial pressure of oxygen (PaO_2_)/inspired oxygen concentration (FiO_2_) ≤ 300 mmHg
Critical	Critical	Meet any of the following conditions: 1.Respiratory failure occurs and requires mechanical ventilation. 2.Shock. 3.With other organ failure and requires ICU monitoring and treatment

**Table 2:** The patient’s ward

Ward	Male (cases)	Female (cases)	Age (years)
**The first infectious disease department, ward 4 (A4)**	15	23	55.50 ± 14.16
**The first infectious disease department, ward 8 (A8)**	29	23	58.01 ± 13.06
**The first infectious disease department, ward 13 (A13)**	24	22	64.56 ± 14.01
**The second infectious disease department, ward 1 (B1)**	36	30	69.35 ± 16.85

All patients were diagnosed in accordance with Diagnosis and Treatment Protocol for COVID-19 (Trial Version 7) and met the Diagnosis and Treatment Protocol for novel coronavirus nucleic acid test and/or serum antibody test of a pharynx swab, as well as pulmonary CT scan images showing new-onset virulent pneumonia [19]. Patients with incomplete clinical data were excluded.

Among the COVID-19 patients who met the diagnostic criteria of the 2018 Chinese guidelines for the management of hypertension [18], 67 patients were included (14 in A4, 13 in A8, 24 in A13 and 16 in B1), accounting for 33.17% of the total number of patients in A4, A8, A13 and B1.Among 67 patients, there were 33 males and 34 females.

Out of the 67 patients, patients who received either an ACEI or ARBs were classified in group A, while patients who did not receive either of them were categorized in group B. There were 22 patients in group A (10 male and 12 female), and 45 patients in group B (male 23, female 22).

### Descriptive data

We observed no significant difference between groups A and B in the general condition of the patients on the day of admission to the hospital (*p* > 0.05) (Tables 3 and 4).

**Table 3:** Comparison in general parameters between groups A and B

Parameters	Group A (***n*** = 22)	Group B (***n*** = 45)	statistical value	***P***
**General information**				
Male (cases)	10	23	0.189^a^	0.664
Female (cases)	12	22	–	–
Age (years)	63.36 ± 12.42	65.33 ± 12.31	0.613^b^	0.542
**Severity classification of COVID-19**			481.5^c^	0.415
Mild (cases)	7	19	–	–
Severe (cases)	14	25	–	–
Critical (cases)	1	1	–	–
**Comorbidities**				
Diabetes (cases)	5	6	0.950a	0.330
Coronary heart disease (cases)	3	7	0.043d	0.836
Cirrhosis (cases)	0	1	*	1.000
Biliary infection (cases)	0	1	*	1.000
Hypothyroidism (cases)	0	1	*	1.000
Alzheimer’s disease (cases)	0	1	*	1.000
Nasopharyngeal carcinoma (cases)	0	1	*	1.000
**Drugs administered to treat COVID-19**				
Lianhua Qingwen capsule	16	30	0.252^d^	
Moxifloxacin	12	22	0.189^d^	
Arbidol	16	31	0.104^d^	
Traditional Chinese Medicine	21	41	*	

^a^ χ^2^ test. ^b^ Student’s t test. ^c^ Kruskal-Willis test. ^d^ continuous correction χ^2^ test. * Fisher’s exact test

**Table 4:** Comparison of clinical parameters between groups

Parameters	At admission	At discharge	statistical value	***p***
Group A: ALT (U/L)	35.05 ± 19.17	32.50 ± 24.72	1.571	0.116
Group B: ALT (U/L)	34.75 ± 22.93	31.13 ± 17.62	−1.220	0.222
statistical value	−0.454^a^	−0.220^a^	–	–
P	0.650	0.826	–	–
Group A: Cr (μmol/L)	68.50 ± 14.53	67.14 ± 1	−0.114	0.909
Group B: Cr (μmol/L)	75.26 ± 20.54	72.00 ± 13.63	−0.488	0.626
statistical value	−0.189^a^	−1.130^a^	–	–
P	0.276	0.183	–	–
Group A: CK (U/L)	49.68 ± 25.61	49.95 ± 15.35	−0.406	0.685
Group B: CK (U/L)	56.98 ± 32.40	55.51 ± 19.03	−0.887	0.375
statistical value	−0.361^a^	−1.030^a^	–	–
P	0.718	0.303	–	–
Group A:Hospitalization time (days)	27.41 ± 6.43	–	–	–
Group B:Hospitalization time (days)	30.07 ± 8.92	–	–	–
statistical value	1.246^b^	–	–	–
P	0.217	–	–	–

^a^ Wilcoxon rank-sum test. ^b^ Student’s t test

The drugs administered to the patients in groups A and B to treat COVID-19 are detailed in Table 2. We observed no significant difference between the two groups in the treatment of COVID-19 (*P* > 0.05) (Table 3).

### Outcome data

The hypertension was controlled in all the patients, and the blood pressures were in the expected range (A related statement is already mentioned above, consider deleting either of the statements). During the treatment, one patient in group A and three patients in group B progressed from the mild pneumonia stage to severe pneumonia stage. However, at the end of their stay in the hospital, all the patients recovered and tested negative for COVID-19 by nucleic acid detection test. No patients had the recurrence of COVID-19 induced pneumonia after discharge. Moreover, there was no significant difference in the general conditions between the two groups at discharge (*p* > 0.05). Although the length-of-stay in the hospital was shorter in group A than that of group B, the difference was not statistically significant (*p* > 0.05). The detailed data are presented in Tables 3 and 4.

We observed no significant difference in the expression levels of ALT, LDH, CK, and Cr between the day of admission and day of discharge in groups A and B (p > 0.05, as shown in Table 4).

## Discussion

In December 2019, the outbreak of COVID-19 began in Wuhan city of Hubei Province in China. Although the epidemic has been controlled to a great extent in China, the disease is still prevalent around the world and is the focus of physicians worldwide. During the early stages of the COVID-19 outbreak, many doctors noticed that the dead victims were often elderly patients with multiple complications such as acute respiratory distress syndrome [20] and hypertension.

Some researchers have proposed that administration of hypertension medication such as ACEIs or ARBs to COVID-19 patients can aggravate the existing disease (COVID-19) [21], or increase the chances of healthy patients encountering COVID-19. Therefore, they advocated for discontinuation/cautious use of such drugs in patients with COVID-19 that down-regulate the renin-angiotensin-aldosterone system (RAAS) system. Despite some recent studies [22], there is currently no confirmatory evidence to support the use of RAS inhibitors in COVID-19 patients. ACEI/ARB drugs should be used in hypertensive patients with stable COVID-19, or hypertensive patients who are at risk of developing COVID-19, as recommended in the 2018 ESC/ESH hypertension guidelines [23]. There is a need to further assess the impact of hypertension and antihypertensive drugs, especially RAS inhibitors, on the course of COVID-19. As a result, some physicians in our hospital also stopped the use of ACEIs and/or ARBs drugs in COVID-19 patients.

Recent studies have demonstrated that classical ACE/Ang-II/AT1R and newly discovered ACE2/Ang 1–7/Mas pathways play a balancing role in the RAAS system. Some studies showed that ARBs or ACEIs induces the expression of ACE2 [15]; however, whether the upregulated expression of ACE2 causes an increased risk of infection with SARS-CoV-2 and/or a deterioration in the progression of COVID-19, is unknown. According to the results of this study and other clinical data from Wuhan [24], there is no significant difference in prevalence of hypertension between un-infected and COVID-19 infected patients. Thus, hypertension and the use of ARBs/ACEI drugs may not be the predisposing factors for COVID-19, although this statement needs further clinical or epidemiological verification. The data collected in this study were from Raytheon Mountain Hospital in Wuhan, which specializes in the treatment of COVID-19. Out of 202 patients in 4 wards, COVID-19 and hypertension were found to co-occur in 33.17% of the patients, which is close to the prevalence of hypertension in China [18].

Compared with the condition of COVID-19 patients who did not receive ACEI or ARB, condition of the patients who received ACEI or ARB did not deteriorate; no increase in death, recovery, or length of stay was observed. The patients who did not receive ACEI or ARB did not see any other therapeutic benefit in their condition of not receiving ACEI or ARB, suggesting that ACEI or ARB did not worsen the condition of COVID-19 patients. Although the pulmonary CT changes of patients in each group were not analyzed in this study, clinical prognosis suggested that the use of ACEI or ARB would not promote the secondary spread of the virus in the lungs of patients, or the possibility of secondary infection was low. Whether the use of ACEI or ARB can aggravate the pneumonia-induced damage in COVID-19 patients is a major point of contention in the use of RAS inhibitors in COVID-19 patients.

Most studies investigating whether ARBs/ACEIs aggravate COVID-19 induced pneumonia have indicated that inhibition of the RAAS system upregulates the expression of ACE2 and alleviates viral pneumonia [14]. On both axes of the RAAS system, the consequence of the ACE/Ang II/AT1R axis causes hypertension and exacerbates lung injury, while it is counteracted by the ACE2/Ang 1–7/MasR axis [12, 13], protecting the lung function [25, 26]. The clinical examination of patients from Wuhan with COVID-19 induced pneumonia showed that IL-1b, IFN-, IL-10, and other inflammatory factors were significantly increased when patients were admitted to the hospital, and the expression level of G-CSF, IP-10, MCP-1, and TNF were upregulated in severe COVID-19 patients. This suggests that there is a correlation between the cytokines/inflammatory factors and the severity of the disease [27]. The mechanism underlying this phenomenon is that COVID-19 downregulated the expression of ACE2, causing an imbalance of ACE/ACE2 and an absolute or a relative increase in the Ang II expression level. This not only induces cytokine storms and systemic inflammatory responses in the patients but also exacerbates inflammatory exudation in the lung [28, 29]. The animal experiments have confirmed that the inflammatory response in the lung could be reversed by increasing the expression level of ACE2 and Ang 1–7 [26, 30].

According to the two functional axes of the RAAS system, ARBs block the interaction of Ang II with its receptor and increase the expression of ACE2. This increases the expression level of Ang1–7 and protects the patients from hypertension and inflammatory injury. ACEI inhibitors block the conversion of Ang I to Ang II. Although the expression of ACE2 is increased, it is not sure whether the Ang1–7 expression level would be upregulated because of the insufficient substrate Ang II. Previous experiments have indicated that blocking the Ang II receptor ATIR better attenuates inflammation and acute lung injury as compared with inhibiting ACE [31]. This study enhanced the question about the application of ACEI drugs, but accurate animal experiments are required to confirm this hypothesis. Similar to the invention of anti-HIV drug Nvevir peptide [32], the discovery of ACE2/Ang 1–7/MasR axis is recognized as a major breakthrough for the discovery of antihypertensive drugs and the treatment of viral pneumonia, including COVID-19 induced pneumonia. Researchers are further investigating this axis to develop effective therapeutic solutions to treat the COVID patients [14, 33–36].

ARBs/ACEIs do not increase the risk of aggravation of COVID-19, but instead helps in treating COVID-19 induced pneumonia. We found that the ABRs/ACEI treated patients stayed shorter in the hospital than the group b during COVID-19 treatment. Although this study might suffer from the limitation of a smaller sample size number of patients, it still provides evidence that ACEs/ARBs do not increase the aggravation risk of COVID-19 pneumonia. To treat hypertension patients with COVID-19 induced pneumonia, anti-hypertensive drugs such as ACEs and ARBs may be used in accordance with the relative guidelines. The treatment regimen with these drugs does not need to be adjusted for the COVID-19 patients.

This study has some other limitations: patients who were given ARBs and ACEI were categorized into the same group because of the limited sample size and time constraints, this study is a retrospective study involving patients in a single hospital. Prospective studies with a larger sample size are warranted. Since all data were collected during the patient’s hospitalization, some comorbidities may not be documented in the clinical data.

## Conclusions

In conclusion, RAS inhibitors such as ACEIs and ARBs have no adverse effect on the clinical prognosis of COVID-19 patients with hypertension.

## Data Availability

The datasets used and analyzed during the current study are available from the corresponding author on reasonable request.

## Abbreviations

ACEI: Angiotensin-Converting Enzyme Inhibitor
ARBs: Angiotensin Receptor Blockers
COVID-19: Coronavirus Disease 2019
ALT: Alanine Aminotransferase
Cr: Serum Creatinine
CK: Creatine Kinase
SARS: Severe Acute Respiratory Syndrome
ACE: Angiotensin-Converting Enzyme
RAAS: Renin-Angiotensin-Aldosterone System
AngII: Angtensin II
SPSS: Statistical Package for the Social Sciences

## References

[1] Lake MA (2020). What we know so far: COVID-19 current clinical knowledge and research. Clin Med (Lond)..

[2] Velavan MCG (2020). The COVID-19 epidemic. Trop Med Int Health..

[3] Wu F, Zhao S, Yu B, Chen Y, Wang W, Hu Y, et al. Complete genome characterisation of a novel coronavirus associated with severe human respiratory disease in Wuhan, China. bioRxiv. 2020. 10.1101/2020.01.24.919183.

[4] Zhou P, Yang X, Wang X, Hu B, Zhang L, Zhang W, et al. Discovery of a novel coronavirus associated with the recent pneumonia outbreak in humans and its potential bat origin. bioRxiv. 2020. 10.1101/2020.01.22.914952.

[5] Lu R, Zhao X, Li J, Niu P, Yang B, Wu H (2020). Genomic characterisation and epidemiology of 2019 novel coronavirus: implications for virus origins and receptor binding. Lancet..

[6] Gorbalenya AE, Baker SC, Baric RS, et al. Severe acute respiratory syndrome-related coronavirus: the species and its viruses -a statement of the coronavirus study group. bioRxiv. 2020. 10.1101/2020.02.07.937862.

[7] World Health Organization. WHO Director-General’s remarks at the media briefing on 2019-nCoV on 11 February 2020. 2020. https://www.who.int/dg/speeches/detail/who-director-general-s-remarks-at-the-media-briefing-on-2019-ncov-on-11-february-2020. Accessed 15 Apr 2020.

[8] Du L, He Y, Zhou Y, Liu S, Zheng B, Jiang S (2009). The spike protein of SARS-CoV-a target for vaccine and therapeutic development. Nat Rev. Microbiol..

[9] Xu X, Chen P, Wang J, Feng J, Zhou H, Li X (2020). Evolution of the novel coronavirus from the ongoing Wuhan outbreak and modeling of its spike protein for risk of human transmission. Sci China Life Sci..

[10] Patel VB, Zhong JC, Grant MB, Oudi GY (2016). Role of the ACE2/Angiotensin 1–7 Axis of the Renin-Angiotensin System in Heart Failure. Circ Res..

[11] Hamming I, Cooper ME, Haagmans BL, Hooper NM, Korstanje R, Osterhaus AD (2007). The emerging role of ACE2 in physiology and disease. J Pathol..

[12] Santos RAS, Sampaio WO, Alzamora AC, Motta-Santos D, Alenina N, Bader M (2018). The ACE2/Angiotensin-(1–7)/MAS Axis of the Renin-Angiotensin System: Focus on Angiotensin-(1–7). Physiol Rev..

[13] Imai Y, Kuba K, Rao S, Huan Y, Guo F, Guan B (2005). Angiotensin-converting enzyme 2 protects from severe acute lung failure. Nature..

[14] Wu Y (2020). Compensation of ACE2 Function for Possible Clinical Management of 2019-nCoV-Induced Acute Lung Injury. Virol Sin..

[15] Ferrario CM, Jessup J, Chappell MC, Averill DB, Brosnihan KB, Tallant EA (2005). Effect of angiotensin-converting enzyme inhibition and angiotensin II receptor blockers on cardiac angiotensin-converting enzyme 2. Circulation..

[16] Fang L, Karakiulakis G, Roth M (2020). Are patients with hypertension and diabetes mellitus at increased risk for COVID-19 infection?. Lancet Respir Med..

[17] Jin YH, Cai L, Cheng ZS, Cheng H, Deng T, Fan YP (2020). A rapid advice guideline for the diagnosis and treatment of 2019 novel coronavirus (2019-nCoV) infected pneumonia (standard version). Mil Med Res..

[18] Writing Group of 2018 Chinese Guidelines for the Management of Hypertension, Chinese Hypertension League, Chinese Society of Cardiology, Chinese Medical Doctor Association Hypertension Committee, Hypertension Branch of China International Exchange and Promotive Association for Medical adn Health Care, Hypertension Branch of Chinese Geriatric Medical Associarion (2018). 2018 Chinese guidelines for the management of hypertension. Chin J Cardiovasc Med.

[19] National Health Commission & State Administration of Traditional Chinese Medicine. Diagnosis and Treatment Protocol for COVID-19 (Trial Version 7). 2020. http://en.nhc.gov.cn/2020-03/29/c_78469.htm. 10.1097/IM9.0000000000000022 PMC7227203 40479241

[20] Yang X, Yu Y, Xu J, Shu H, Xia J, Liu H (2020). Clinical course and outcomes of critically ill patients with SARS-CoV-2 pneumonia in Wuhan, China: a single-centered, retrospective, observational study. Lancet Respir Med..

[21] Diaz JH (2020). Hypothesis: angiotensin-converting enzyme inhibitors and angiotensin receptor blockers may increase the risk of severe COVID-19. J Travel Med.

[22] ESH Statement on COVID-19: Statement of the European Society of Hypertension (ESH) on hypertension, Renin Angiotensin System blockers and COVID-19.April 15th 2020. 2020. https://www.eshonline.org/esh-content/uploads/2020/06/Statement-ESH-on-Hypertension-RAS-Blockers-and-COVID-19-Update-April-15-2020.pdf. Accessed 11 Feb 2020.

[23] Williams B, Mancia G, Spiering W, Rosei EA, Azizi M, Burnier M (2018). 2018 ESC/ESH Guidelines for the Management of Arterial Hypertension: The Task Force for the Management of Arterial Hypertension of the European Society of Cardiology and the European Society of Hypertension: The Task Force for the Management of Arterial Hypertension of the European Society of Cardiology and the European Society of Hypertension. J Hypertens.

[24] Guan WJ, Ni ZY, Hu Y, Liang WH, Ou CQ, He JX (2020). Clinical characteristics of coronavirus disease 2019 in China. N Engl J Med..

[25] Ye R, Liu Z (2020). ACE2 exhibits protective effects against LPS-induced acute lung injury in mice by inhibiting the LPS-TLR4 pathway. Exp Mol Pathol..

[26] Wösten-van Asperen RM, Lutter R, Specht PA, Moll GN, van Woensel JB, van der Loos CM (2011). Acute respiratory distress syndrome leads to reduced ratio of ACE/ACE2 activities and is prevented by angiotensin-(1–7) or an angiotensin II receptor antagonist. J Pathol..

[27] Huang C, Wang Y, Li X, Ren L, Zhao J, Hu Y (2020). Clinical features of patients infected with 2019 novel coronavirus in Wuhan, China. Lancet.

[28] Kuba K, Imai Y, Rao S, Gao H, Guo F, Guan B (2005). A crucial role of angiotensin converting enzyme 2 (ACE2) in SARS coronavirus-induced lung injury. Nat Med..

[29] Yang P, Gu H, Zhao Z, Wang W, Cao B, Lai C (2014). Angiotensin-converting enzyme 2 (ACE2) mediates influenza H7N9 virus-induced acute lung injury. Sci Rep.

[30] Zambelli V, Bellani G, Borsa R, Pozzi F, Grassi A, Scanziani M (2015). Angiotensin-(1–7) improves oxygenation, while reducing cellular infiltrate and fibrosis in experimental Acute Respiratory Distress Syndrome. Intensive Care Med Exp..

[31] Wösten-van Asperen RM, Lutter R, Specht PA, Specht PA, van Woensel JB, van der Loos CM (2010). Ventilator-induced inflammatory response in lipopolysaccharide-exposed rat lung is mediated by angiotensin-converting enzyme. Am J Pathol..

[32] Wild CT, Shugars DC, Greenwell TK, McDanal CB, Matthews TJ (1994). Peptides corresponding to a predictive alpha-helical domain of human immunodeficiency virus type 1 gp41 are potent inhibitors of virus infection. Proc Natl Acad Sci U S A..

[33] Chen Y, Guo Y, Pan Y, Zhao ZJ (2020). Structure analysis of the receptor binding of 2019-nCoV. Biochem Biophys Res Commun.

[34] Gurwitz D. Angiotensin receptor blockers as tentative SARS-CoV-2 therapeutics. Drug Dev Res. 2020. 10.1002/ddr.21656. 10.1002/ddr.21656 PMC7228359 32129518

[35] Tan WSD, Liao W, Zhou S, Mei D, Wong WF (2018). Targeting the renin-angiotensin system as novel therapeutic strategy for pulmonary diseases. Curr Opin Pharmacol..

[36] Huentelman MJ, Zubcevic J, Hernández Prada JA, Xiao X, Dimitrov DS, Raizada MK (2004). Structure-based discovery of a novel angiotensin-converting enzyme 2 inhibitor. Hypertension..

